# Tomographic analysis of relationship of mandibular morphology and third molars eruption

**DOI:** 10.1186/s12903-023-03653-0

**Published:** 2023-11-23

**Authors:** Jéssica de Fátima Segantin, Gabriela Barbosa Bisson, Letícia Liana Chihara, Osny Ferreira Júnior

**Affiliations:** Department of Surgery, Stomatology, Pathology and Radiology, Bauru School of Dentistry. University of São Paulo, Alameda Dr. Octávio Pinheiro Brisolla, 9-75, Bauru, SP 17012-901 Brazil

**Keywords:** Tomography, Third molar, Mandible, Tooth eruption

## Abstract

**Background:**

Lower third molars (L3M) are the last teeth to erupt in the oral cavity. Uneruption of these teeth still raises questions about its causes, in the literature (1) genetic factors, (2) dental lamina activity and, mainly, (3) insufficient growth and development of the bone bases are included. While the lack of space theory influenced by mandibular morphology and size of L3M was argued to be the main reason for L3M impaction, there is a limitation in the literature in examining such association using more accurate tomographic analysis obtained from CBCT. This work aimed to evaluate the relationship between mandibular morphology and the eruption of L3M.

**Methods:**

In this regard, 85 Cone Beam Computed Tomographies (CBCT), with 147 L3M, were selected from the archives of the Department of Surgery, Stomatology, Pathology and Radiology, Bauru School of Dentistry, obtained using an Accuitomo® Morita device and using the Dolphin Imaging 11.9 software. L3M eruption was related to linear measurements of jaw length (Co-Gn), retromolar space dimension (D2R), mesiodistal width of the L3M crowns, mandibular first molars (L1M) and mandibular canines (LC) and the angle mandibular (Ar-Go-Me). Independent samples t-test, chi-square tests and logistic regression were performed adopting a significance level of 5%.

**Results:**

The average mandible length of 116.446 mm + 6.415 mm, retromolar space of 11.634 mm + 2.385 mm, mesiodistal size of the L3M of 10.054 mm + 0.941 mm, sum of the mesiodistal widths of the L1M and LC of 15.564 mm + 1.218 mm and mandibular angle of 127.23° + 6.109. There was no statistically significant association between these factors and the eruption.

**Conclusion:**

With the results obtained in this study, we conclude that the length and angle of the mandible, teeth size and dimension of the retromolar space are not associated with the L3M eruption.

## Introduction

Third molars are the last teeth to erupt in the oral cavity, with the lower teeth having the highest uneruption rate (17–69%) when compared to other teeth [1].

Inadequate space associated with craniofacial development and mandibular morphology are considered the main causes of uneruption [1-3]. However, other theories such as: eruption in the distal direction or lack of eruptive strength, early physical maturity, delayed mineralization and difficulties related to the peculiarities and morphological variations of L3 are also found in the literature [4-6].

The age at which lower third molars erupt may be influenced by racial variation, nature of the diet, degree of use of the masticatory apparatus and genetic inheritance [7]. According to Cortella, Shofer and Ghafari [8] during puberty, the growth of bone bases and structural transformations are evident. At this stage, the growth of the width of the mandible is completed, leaving only sagittal and vertical development [8].

The mechanisms that determine the uneruption of L3M are still obscure [9]. Most researchers claim that it is the result of a lack of space in the mandible, but they disagree about the primary mechanism: mandibular body width, mandibular angle, timing and mandibular growth in relation to eruption or a combination of these factors [9].

The different morphological characteristics of the L3M, irregular size, root dilacerations and variable position in the mandible, are also associated in the literature with the high rate of uneruption of these teeth [6]. According to Hattab [10] and Vranckx et al. [11] severely angulated third molars have a minimal chance of future eruption and a greater risk of developing a relationship with the mandibular canal, even with adequate retromolar space.

Clinically, the uneruption of the teeth can generate painful symptoms, pericoronitis, trismus, resorptions and periodontal problems in the second molars [12]. To avoid these situations, the evaluation of the dental surgeon is fundamental in indicating the surgical removal of the L3M [12].

Most of the research addressing uneruption of L3M has been carried out using 2D imaging exams, such as panoramic radiography [13, 14]. However, with CBCT it is possible to perform evaluations of the region of interest in the three planes of space, promoting greater accuracy of measurements [15].

In our search in the scientific literature, no studies were found associating various aspects of mandibular morphology and the size of the teeth with the eruption of the L3M in detail, using computed tomography. Therefore, the aim of this work is to analyze the relationship between mandibular morphology and tomographic L3M eruption, using CBCT and Dolphin Imaging software.

## Materials & methods

### Sample size calculation

Based on the proportion of erupted and unerupted teeth found in the literature and in the pilot study, a sample calculation was carried out according to Peduzzi et al. [16] and Hsieh [17] with the aid of an online sample size calculator (available at: http://estatistica.bauru.usp.br/calculoamostral/ta_diferenca_media_independente.php).

The calculation to define the sample required for the multiple logistic regression analysis was carried out considering the number of independent variables [5] and and the lowest expected proportion (35% erupted). The required sample size was 143.

For the independent samples t-test, the highest standard deviation among the variables analyzed was considered to be 0.89 (Co-Gn), resulting in a necessary N of 51.

### Materials

This retrospective study was carried out with CBCT performed between 2015 and 2018 selected from the archives of Radiology Clinic of Bauru School of Dentistry, University of São Paulo, after approval by the Research Ethics Committee, from the same institution. Images were taken with Accuitomo® 170 3D tomograph (J. Morita, United States of America) with FOV (Field of View) of 170 × 120 cm, 80 kVp, 2mAs, acquisition time of 40 seconds and voxel of 80 μm.

The study included patients over 17 years of age, with at least L3M erupted or not and L2M, L1M, LC and lower central incisor (LCI), with intact crowns on the same side of the mandible.

Patients with alterations in the mandible, such as cysts and tumors, fractures, cleft lip and palate, and those whose CT scans had distortions and did not show the complete condyles and chin were excluded.

The studied variables were mandible length, mandibular angle, retromolar space size and teeth size. These measurements were obtained from the sagittal reformats of the Dolphing Imaging software version 11.9.

### Methods

**Fig. 1:**
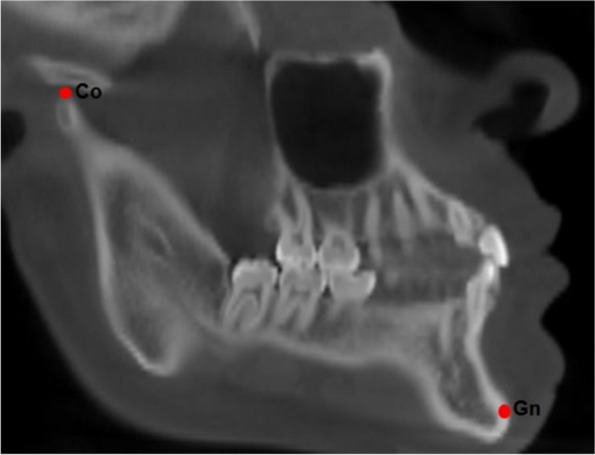
Effective mandible length with Co-Gn points

To measure the effective length of the mandible (Co-Gn), the distance between the cephalometric points was used, demarcating the condyle (Co) corresponding to the most superior and posterior point of the mandibular condyle until the Gnatium (Gn) being the lowest point and anterior of the mandible according with Mc Namara Jr [18] (Fig. 1).

**Fig. 2:**
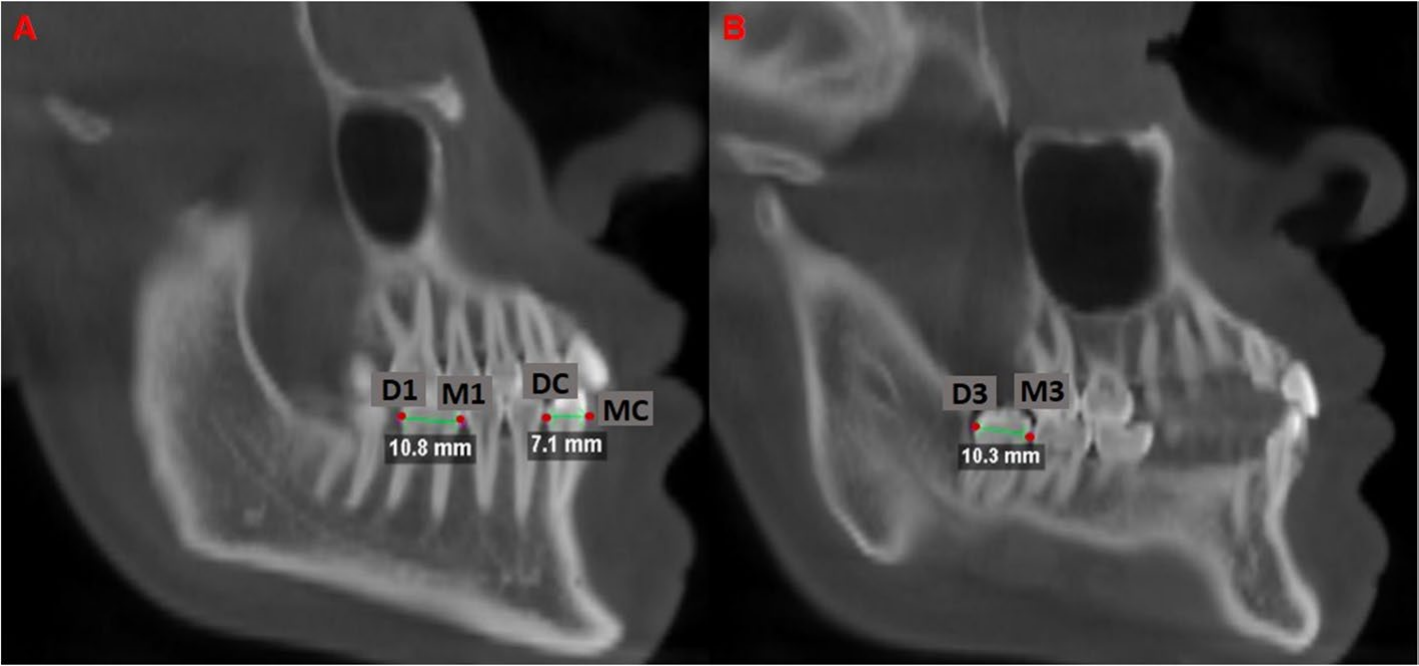
Size of teeth **A**) Measurement of lower first molars (L1M) and canines (LC), **B**) Measurement of lower third molars (L3M)

Then, a sagittal reformat was created in order to pass through the LC, L1M and L3M to measure the size of the teeth corresponding to the mesiodistal width at the level of the prosthetic equator. For each tooth, the most mesial point and the most distal point was marked, on the prosthetic equator being for the canine MC-DC, for the L1M corresponding to the points M1-D1 and for the L3M the points M3-D3 (Fig. 2).

It is noteworthy that, in 62 CBCT that had bilateral L3M, the measurements were taken with the points positioned to each side (right and left). For the other 23 CBCT, with unilateral L3M, only the side of interest was measured.

To determine the eruption or not of the L3M, the L3M whose occlusal face was at the same level as the occlusal face of the L2M, that is, in a vertical position, were considered erupted. Other situations were classified as unerupted.

**Fig. 3:**
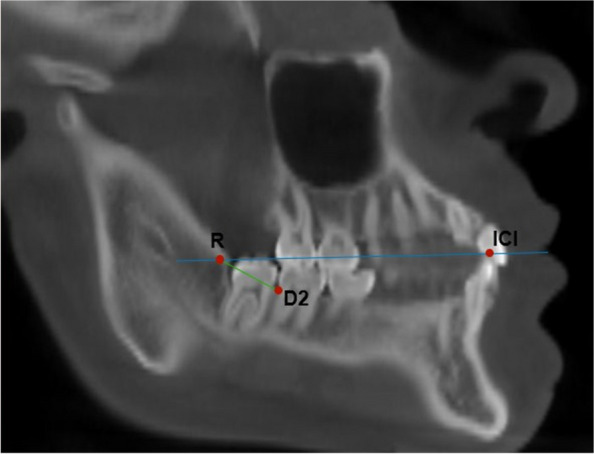
Measurement of retromolar space (D2-R)

As for the retromolar space size (D2-R), the D2 point corresponding to the most distal point of the cementoenamel junction of the L2M was demarcated and the R point determined by the crossing of the occlusal plane, formed by the straight line that passes through the higher point of the LC and L2M cusps, with the anterior edge of the mandibular ramus. Thus, the retromolar space (D2-R) was measured by the distance from point D2 to point R (Fig. 3).

**Fig. 4:**
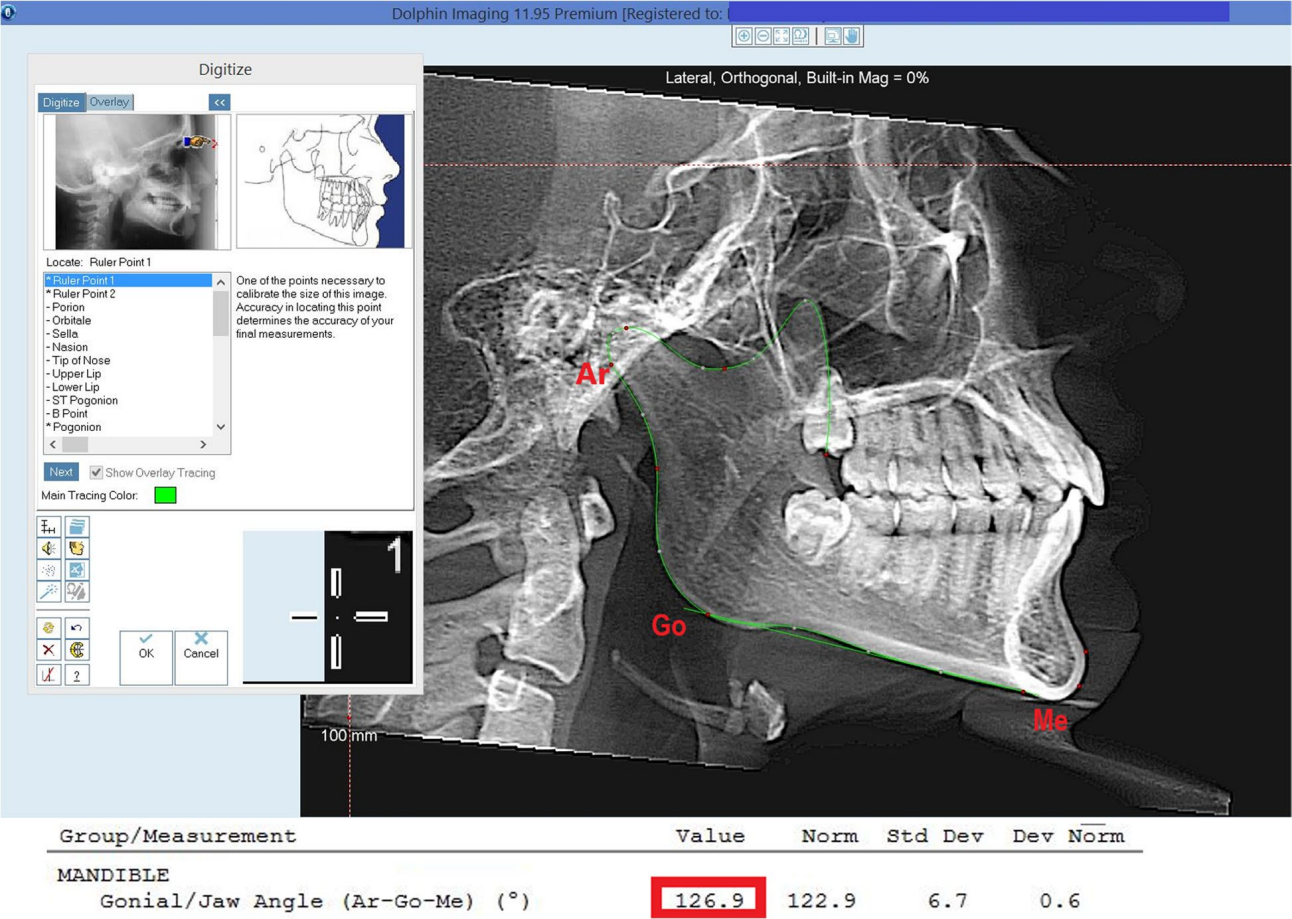
Marking the points of interest in sagittal reformatting: Ar-Go-Me and the gonial angle measurement provided by the software

For the mandible angle, the following points were marked: Articulare (Ar), Gonio (Go) and Menton (Me), according with Rodriguez-Cardenas et al. [19], Naqvi [20] and Zhang et al. [21]. The software automatically traces the angle according to the Jarabak analysis (Fig. 4).

### Statistical analysis

Initially, measurements performed in 30 CBCT, repeated with an interval of 15 days, were used to calculate method error and intra-examiner confidence and sample calculation.

Independent samples t-test, chi-square tests and logistic regression tests were performed,the dependent variable was the eruption of L3M and the independent variables were gender, mandibular length, retromolar space and tooth size. In all tests, a significance level of 5% was developed to analyze differences between variables.

All tests were performed with the *SPSS V20 program (IBM Corp. Released 2011. IBM SPSS Statistics for Windows, Version 20.0. Armonk, NY: IBM Corp.) e Minitab 16 (Minitab 16 Statistical Software 2010. Computer software. State College, PA: Minitab, Inc.).*

## Results

### Sample description

**Fig. 5:**
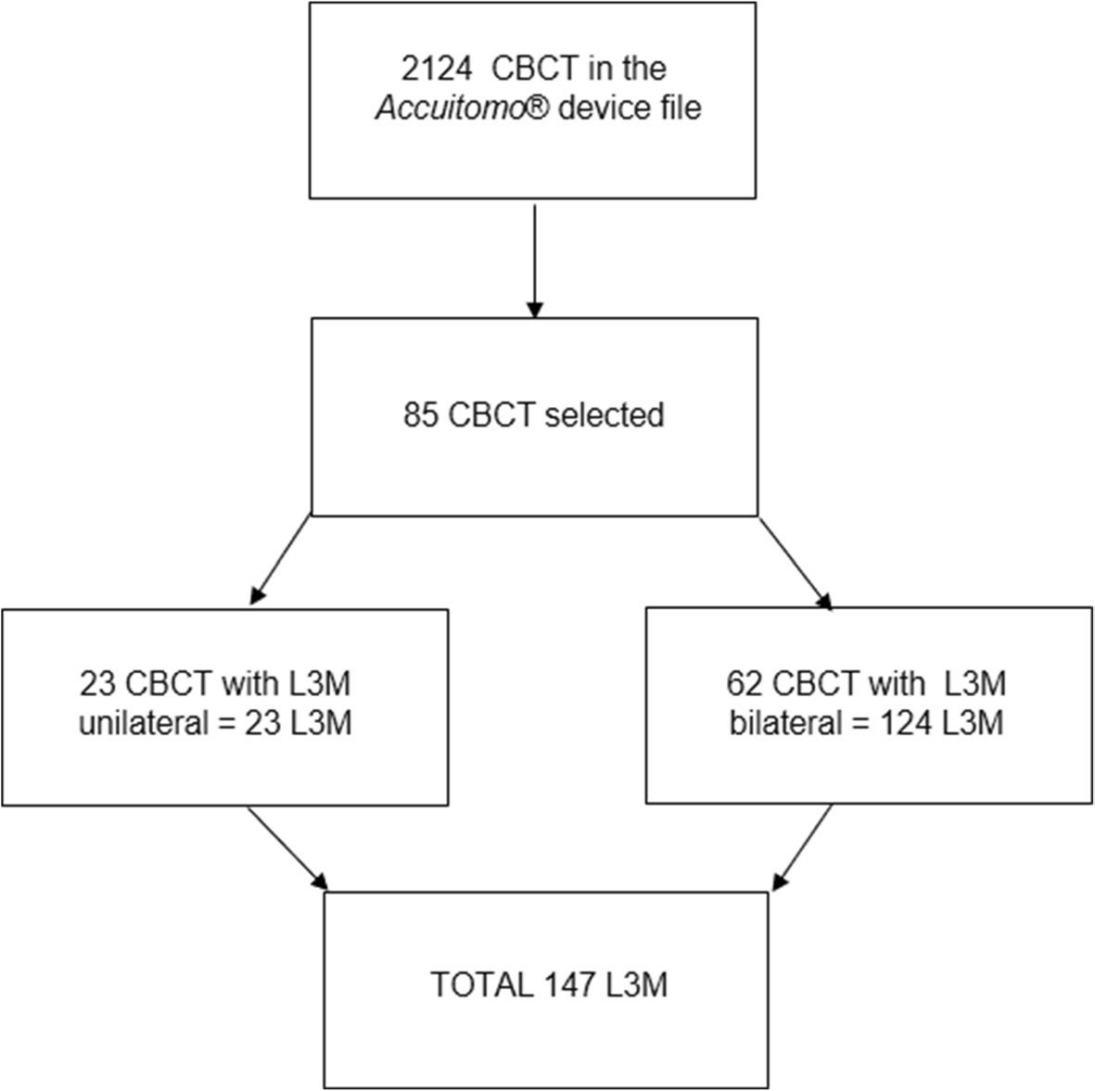
CBCT selection flowchart

Following the inclusion criteria, 85 CBCT were evaluated, with 147 hemi-mandibles with L3M (Fig. 5), with a mean age of 22.35 years, ranging between 17 and 32 years. More than half of the sample (53.1%) was composed of male patients and 46.9% of female patients.

### Relationship between the variables and the outbreak of L3M

Using the Chi-square test, we sought to identify whether there is a relationship between gender and the eruption rate, however, *p* = 0.33 indicated that there was no statistically significant difference.

The results of the measurements taken, considering erupted and unerupted teeth, are shown in Table 1. The T-test performed shows the relationship between these measurements and the eruption of the L3M.

**Table 1:** Mean and standard deviation of linear measurements and relation to L3M eruption

		Erupted L3M	Unerupted L3M	***p*** value
**Age**	N	55	92	
	Mean	23.530	21.650	0.007*
	SD	0.572	0.403	
**Co-Gn (mm)**	N	55	92	
	Mean	115.973	116.728	0.490*
	SD	0.891	0.659	
**LC (mm)**	N	55	92	
	Mean	6.047	6.146	0.430*
	SD	0.099	0.075	
**L1M (mm)**	N	55	92	
	Mean	9.371	9.505	0.310*
	SD	0.098	0.083	
**LC + L1M (mm)**	N	55	92	
	Mean	15.418	15.651	0.263*
	SD	0.153	0.131	
**L3M (mm)**	N	55	92	
	Mean	10.013	10.079	0.679*
	SD	0.138	0.092	
**D2-R (mm)**	N	55	92	
	Mean	1.847	1.419	0.333*
	SD	0.363	0.262	
**Ar-Go-Me**	N	14	35	
	Mean	127.800	126.900	0.628*
	SD	1.932	0.919	

*Independent samples t-test

Furthermore, multiple logistic regression analysis was performed having eruption as dependent variable and gender, Co-Gn distances, D2-R, L3M and teeth size as independent variables, but no statistically significant result was obtained in any of the variables (Table 2). The odds ratio was calculated taking uneruption as a reference and the reference used for the variable gender was female.

**Table 2:** Multiple logistic regression analysis in relation to the eruption of the L3M

Independent variables	Wald	p	OR^a^	CI 95%^b^
Gender (M/F)	0.254	0.614	0.82	0.37–1.78
Co-Gn	2.312	0.128	0.94	0.85–1.02
D2-R	2.007	0.157	1.12	0.95–1.29
L3M width	0.029	0.865	0.97	0.64–1.44
LC + L1M	0.524	0.469	0.89	0.64–1.22
Constant	0.020	0.889	1.72	

^a^*OR* odds ratio

^b^*CI95* 95% confidence interval

For the analysis of the relationship between the angle of the mandible and the eruption of the lower third molars, 51 hemi-mandibles were evaluated. When relating the mandibular angle with the eruption of the L3M, it was possible to observe that the difference is not statistically significant (Table 1).

The result obtained in the logistic regression having eruption as a dependent variable and the mandibular angle as an independent variable, also confirms that the difference is not statistically significant (*p* = 0.97).

## Discussion

The present study was carried out to evaluate the relationship between mandibular morphology and L3M eruption using CBCT and Dolphin Imaging software. The results indicated that the mandibular morphology and the size of the teeth are not associated with the eruption of L3M.

L3M usually erupt between ages 17 and 26, varying considerably between populations and according to gender [7, 22]. In this study, the mean age found was 22 years. The results obtained showed that age is associated with the eruption of L3M, which was expected. Since it influences the process of odontogenesis and consequently the eruption of teeth.

Because they are the last molars to erupt, lack of space is often the justification for these teeth not erupting [22].

Regarding the effective length of the mandible measured in this work by the distance between the point Co and Gn, it was described in the literature by Mc Namara Jr [18]. Used in recent studies such as the one by Santos et al. [8] which standardized the measurements of the dimensions of the maxillomandibular complex in Brazilians, being found for the average of 114.51 mm when combined both genders. These values corroborate those of this study, with an average mandibular length of 116.446 mm. Different from the results of Olayemi [23], who calculated the mandibular length of the Nigerian population with the same reference points, but found higher values. As these are different countries, the ethnicity of the population becomes a determining factor to explain these differences found.

Olayemi [23] also sought to analyze whether the size of other teeth in the arch influences the L3M eruption rate. However, like this study, the size of the teeth did not influence the eruption of the L3M.

To evaluate the relationship between the L3M size and the eruption index, its mesiodistal length was measured separately. However, the mean size obtained for erupted and unerupted L3M was similar.

The lack of space between the second permanent molar and the mandibular ramus was identified as an important factor in the etiology of uneruption of the L3M [24]. In the study by Aliaga-Del Castillo et al. [25], the size of the retromolar space, measured in the most posterior region of the mandible, was presented as a determining factor in the eruption or not of the L3M. In this study, contrary to expectations, the retromolar space did not influence the L3M eruption.

Begtrup et al. [26] sought to relate, through panoramic radiographs, the size and mandibular angle with the rate of eruption of the L3M, according to the authors, the longer the mandible, the greater the chance of eruption of these teeth, however, no relationship was found with the mandibular angle. The results obtained in this study with CBCT indicated that both the mandibular length and the mandibular angle did not affect the eruption of the L3M.

One of the hypotheses would be that the mandibular angle would interfere with the L3M eruption, as the result of Al-Gunaid et al. [27], but in this study the average between the mandibular angle in erupted and unerupted teeth was similar, contradicting this hypothesis.

The study by de Mayama et al. [28] corroborates the findings of this research, in which the average value of the mandibular angle found for patients without skeletal alterations or congenital absence of teeth was 127.8°.

As we can see, the vast majority of studies on the subject were performed with panoramic radiographs and have divergent results in the literature. Panoramic radiography is a two-dimensional image that has distortions inherent to image formation [29, 30]. However, several studies show that the three-dimensional image is more accurate, reliable and ensures surgical planning with greater predictability [29-33].

A limitation of the study is a relatively small sample, this was because all CT scans were performed in a single center. This study showed that mandibular morphology and tooth size are not determinant in the eruption of L3M.However, future studies must be carried out to try to determine other factors that may influence this process.

## Conclusion

With the results obtained in this study, we conclude that the length and angle of the mandible, teeth size and dimension of the retromolar space are not associated with the L3M eruption.

## Data Availability

The datasets used and/or analysed during the current study are available from the corresponding author on reasonable request.

## Abbreviations

Ar: Articulare
CBCT: Cone Beam Computed Tomographies
CI: Confidence interval
Co: Condyle
CT: Computed tomography
DC: Canine distal
D1: First molar distal
D3: Third molar distal
D2R: Retromolar space dimension
FOV: Field of View
Go: Gonion
Gn: Gnatium
kVp: Kilovoltage to Peak Power
L1M: Lower first molars
L2M: Lower second molar
L3M: Lower third molar
LC: Lower canine
LCI: Lower central incisor
mAs: Milliampere second
MC: Canine mesial
Me: Menton
mm: Millimeter
M1: First molar mesial
M3: Third molar mesial
N: Number
OR: Odds ratio
2D: Two dimensional
3D: Three dimensional
μm: Micrometer
